# MicroRNA‐216a induces endothelial senescence and inflammation *via* Smad3/IκBα pathway

**DOI:** 10.1111/jcmm.13567

**Published:** 2018-03-07

**Authors:** Shujun Yang, Xuenan Mi, Yu Chen, Congrui Feng, Zhihui Hou, Rutai Hui, Weili Zhang

**Affiliations:** ^1^ State Key Laboratory of Cardiovascular Disease FuWai Hospital National Center for Cardiovascular Diseases Peking Union Medical College & Chinese Academy of Medical Sciences Xicheng District Beijing China; ^2^ Beijing Institute for Brain Disorders Center for Brain Disorders Research Capital Medical University Beijing China; ^3^ Department of Radiology FuWai Hospital National Center for Cardiovascular Diseases Peking Union Medical College& Chinese Academy of Medical Sciences Xicheng District Beijing China

**Keywords:** atherosclerosis, endothelial senescence, inflammation, microRNA‐216a, Smad3

## Abstract

Vascular endothelial senescence contributes to atherosclerosis and coronary artery disease (CAD), but the mechanisms are yet to be clarified. We identified that microRNA‐216a (miR‐216a) significantly increased in senescent endothelial cells. The replicative senescence model of human umbilical vein endothelial cells (HUVECs) was established to explore the role of miR‐216a in endothelial ageing and dysfunction. Luciferase assay indicated that Smad3 was a direct target of miR‐216a. Stable expression of miR‐216a induced a premature senescence‐like phenotype in HUVECs with an impairment in proliferation and migration and led to an increased adhesion to monocytes by inhibiting Smad3 expression and thereafter modulating the degradation of NF‐κB inhibitor alpha (IκBα) and activation of adhesion molecules. Conversely, inhibition of endogenous miR‐216a in senescent HUVECs rescued Smad3 and IκBα expression and inhibited monocytes attachment. Plasma miR‐216a was significantly higher in old CAD patients (>50 years) and associated with increased 31% risk for CAD (odds ratio 1.31, 95% confidence interval 1.03‐1.66; *P *= .03) compared with the matched healthy controls (>50 years). Taken together, our data suggested that miR‐216a promotes endothelial senescence and inflammation as an endogenous inhibitor of Smad3/IκBα pathway, which might serve as a novel target for ageing‐related atherosclerotic diseases.

## 1 INTRODUCTION

Cardiovascular diseases present a great public health burden for aged patients and their families. Structural and functional changes of vessels, accumulating throughout the life‐time, increase the risk of cardiovascular diseases. Endothelial cell senescence has been involved in endothelial dysfunction and inflammation which promotes the process of atherogenesis^1^; moreover, vascular endothelial cells with senescent phenotype have been found in the human atherosclerotic plaques.^2^ But, the underlying mechanisms of ageing‐induced attenuation of endothelial functions are not to be fully clarified.

MicroRNAs (miRNAs) are identified as the single‐stranded, small, non‐coding RNAs with approximately 22 nucleotides. The mature miRNAs can repress post‐transcriptional level of gene expression by binding to complementary sequences located on the 3′ untranslated region (3′UTR) of their target specific messenger RNAs (mRNAs).^3^ Increasing evidence suggest a significant role of miRNAs as mediators in vascular ageing and inflammatory process in animal models and human studies.^4^, ^5^ To date, several miRNAs have been identified to regulate the process of endothelial senescence and inflammation, such as miR‐217,^6^ miR‐34a,^7^ miR‐146a^8^, ^9^ and miR‐181b.^10^ Nevertheless, the effect of miRNAs that regulate endothelial cell ageing and inflammation has not been fully clarified.

Recently, through a microarray approach, a particular microRNA (miR‐216a) has been first identified to show a significant differentially expression when compared the miRNA profile obtained in senescent human endothelial cells *versus* young cells.^6^ To date, whether miR‐216a, located on chromosome 2p16.1, plays a key role in endothelial cell ageing remains unclear.

Computational miRNA target analysis from miRNA databases suggests that Smad3 is a potential target gene of miR‐216a. Transforming growth factor beta 1 (TGFβ1) is a pleiotropic growth factor and potentially modulates the function of cells that contribute to atherosclerotic formation, such as vascular smooth muscle cells, endothelial cells, T cells and macrophages. Smad3/IκBα signalling is the main downstream mediator and responsible for the anti‐inflammatory effects of TGFβ1.^11^ The enhancement of Smad3 phosphorylation and nuclear expression can increase NF‐κB inhibitor alpha (IκBα) degradation and nuclear factor‐kappa B (NF‐κB) activation, which thus promotes the inflammatory reaction and atherosclerosis.^12^

In this study, we applied senescent endothelial cell model and for the first time investigated whether miR‐216a can induce endothelial ageing and promote endothelial inflammation by serving as an endogenous inhibitor for the TGFβ1/Smad3 pathway. Furthermore, we performed a large coronary artery disease (CAD) case–control study strictly diagnosed by coronary computed tomography angiography and assessed the association of miR‐216a expression with the risk of CAD, particularly in aged individuals.

## 2 METHODS

### 2.1 Cell culture, miRNA lentiviral infection and miRNA transfection

Primary human umbilical vein endothelial cells (HUVECs) were cultured in endothelial cell medium (ScienCell, San Diego, CA, USA) added with 5% foetal bovine serum and a replicative senescence model was constructed. Population‐doubling levels (PDLs) were numbered during passages according to previous studies.^6^ In brief, the number of population doublings (PDs) was calculated according to the equation PD = log_2_(Ch/Cs), in which Ch is the number of viable cells at harvest and Cs is the number of cells seeded.

To establish stable cell line of miR‐216a, PDL4 HUVECs were infected with pre‐miR‐216a recombinant lentiviruses (Ubi‐EGFP‐MCS‐IRES‐puromycin) and negative control (NC) vectors (Genechem, Shanghai, China), respectively. Three days after infection, cells were treated with 400 ng/mL puromycin (Sigma‐Aldrich, Saint Louis, MO, USA) for selection. Infection efficiency (>95%) was evaluated by enhanced green fluorescent protein (EGFP) florescence analysis. miR‐216 stable line and its negative control were serially passaged, and PDLs were calculated. The PDLs‐versus‐time growth curves were built to assess the replicative potential over time, respectively, for naturally cultured HUVECs and for miR‐216a lentivirus transfected HUVECs.

To explore the regulatory role of miR‐216a on target gene expression, miR‐216a mimics or miR‐216a inhibitors (GenePharma, Suzhou, China) were transfected at a final concentration of 50 nM with the use of lipofectamine 3000 reagent (Invitrogen, Carlsbad, CA, USA). After incubation for 2 days, cells were harvested.

### 2.2 Senescence‐associated β‐galactosidase staining

Cells senescence was assessed by the senescence‐associated β‐galactosidase (SA‐β‐gal) activity *via in situ* staining. Images of the staining were analysed in more than five random microscopic fields per sample and percentages of SA‐β‐gal positive‐staining cells were calculated by counting five fields. The details were described in Data S1.

### 2.3 Assays for endothelial proliferation, migration, angiogenic and cell adhesion activity

The experiments for endothelial functions including cell proliferation, migration, angiogenic and cell adhesion activity were, respectively, performed in PDL8 (at 3 days after transduction of Lv‐miR‐216a) and PDL20 (at about 15 days after transduction of Lv‐miR‐216a). The details were described in Data S1. Briefly, the MTS assay was performed to assess the potential effect of miR‐216a on endothelial proliferation ability. The scratch test was performed to assess the effect of miR‐216a on endothelial migration and wound healing ability. Wound healing rate was calculated: % wound healing = [(Area of original wound‐ Area of wound after healing)/Area of original wound] × 100%. Each wound was analysed in five different areas.

The tube formation assay was performed to study the effect of miR‐216a on angiogenic activity. Photographs of tube morphology were captured in more than five random microscopic fields, and the cumulative mean tube lengths per field of view were quantified. Cell adhesion assay was performed to assess the effect of miR‐216a on endothelial adhesive capacity to monocytes. The non‐adherent THP‐1 cells (China Infrastructure of Cell Line Resources, Beijing, China) were removed by PBS, and the adherent cells were counted in five random fields.

### 2.4 mRNA expression analysis

The mRNA expression of ageing‐related genes p53 and p21, intercellular adhesion molecule 1 (ICAM1) and vascular cell adhesion molecule 1 (VCAM1), and TGFβ1 pathway genes TGFβ1, Smad2, Smad3 and IκBα were analysed by real‐time PCR, respectively. Total RNA of HUVECs was extracted by TRIzol reagent (Invitrogen, Carlsbad, CA, USA); then, cDNAs were synthesized using PrimeScript Reverse Transcriptase assay (Takara, Dalian, China) according to the manufacturer's instructions. Real‐time PCR was performed using SYBR Green qPCR mix (YEASEN, Shanghai, China) on the ABI 7500 System (Applied Biosystems, Foster City, CA, USA). GAPDH levels were used as internal controls, and fold changes were calculated by relative quantification (2^−ΔΔCt^). The primers applied for real‐time PCR were shown in Table S1.

### 2.5 miRNA isolation and expression analysis

Enriched miRNAs were extracted from HUVECs using miRNeasy Mini Kit (QIAGEN, Hilden, Germany). miRNAs in patients' plasma were extracted by miRNeasy Plasma Kit (QIAGEN, Hilden, Germany) with introducing a spiked C.elegans miR‐39 (cel‐39). Real‐time PCR was conducted to examine miR‐216a using miScript II reverse transcription kit and miScript SYBR Green PCR kit (QIAGEN, Hilden, Germany) with ABI 7500 System according to the manufacturer's protocol. The U6 small nuclear RNA was used as internal reference for HUVECs; cel‐39 was applied as external normalized reference for human plasma samples.

### 2.6 Telomerase enzyme activity and telomere length measurement

To assess the telomerase activity during endothelial senescence, real‐time PCR was performed.^13^ Relative mean telomere length in the endothelial cells was determined by a quantitative real‐time PCR‐based method that compares telomere repeat copy number (T) to single‐copy gene copy number β‐globin (S) (T/S ratio).^14^ The details were shown in Data S1.

### 2.7 Plasmid construction and luciferase reporter assay

The Smad3 3′UTR sequence spanning a total of 2002 base pairs (bps) was amplified with primers: forward 5′ CTCAACGCGTGCGTCTGCTCTGGTGGCT 3′ and reverse 5′ GGCGCAAGCTTCACCTGGAGTAAGACACGACTTC 3′, which contains a miR‐216a consensus response element located between 790 and 796 bps of sequence ENST00000327367.4. The amplicon was inserted in the downstream of the firefly luciferase gene in the pMIR‐REPORT™ Luciferase plasmid (Ambion, Austin, TX, USA). Additionally, the Smad3 3′UTR mutant vector containing mutated miR‐216a binding sites was also constructed. The regulatory role of miR‐216a on Smad3 mRNA expression was assessed using luciferase reporter assay in HEK293T cells (China Infrastructure of Cell Line Resources, Beijing, China). The details were described in Data S1.

### 2.8 Western blot analysis

HUVECs were harvested, proteins extracts were isolated, and the effects of miR‐216a on Smad2, Smad3, p65 and IκBα protein levels were determined by Western blot assay. Bands were visualized with FluorChem R, M and E Systems (ProteinSimple, CA, USA) and quantified with AlphaView Software. The details were shown in Data S1.

### 2.9 Subjects for CAD case‐control study

Patients in this study were unrelated and consecutively admitted to FuWai Hospital from May 2016 to May 2017. All subjects provided their written informed consents with the declaration to be of Han nationality. The study was approved by the ethics committee of FuWai Hospital. Inclusion criteria of CAD were defined as having ≥50% narrowing of the lumina of at least 1 of the major coronary arteries by coronary angiography.^15^ The controls were selected from the subjects admitted to FuWai hospital for excluding coronary heart disease, with ≤20% stenosis of major coronary artery and without any vascular disease. All subjects were excluded with a history of other heart diseases including cardiomyopathy, valvular heart disease and congenital heart disease.

The present study comprised 176 CAD patients and 342 age‐matched control subjects, and the clinical features were recorded. Blood samples were collected in the morning after an overnight fast. Plasma biochemical indexes were examined.

### 2.10 Statistical analysis

The one‐sample Kolmogorov‐Smirnov test was used to assess the normality of quantitative variables. Quantitative variables were presented as means ± SD or medians and interquartile ranges (IQRs), and categorical variables as percentages. The χ^2^ test was used to compare qualitative variables. Group differences of quantitative variables were compared by Student's *t* test or one‐way ANOVA as appropriate, and the Mann–Whitney nonparametric test was used to compare variables with non‐normal distributions. Logistic regression models were performed to evaluate odds ratios (ORs) and 95% confidence intervals (CIs) for the association between plasma miR‐216a levels and CAD. Data were analysed by SPSS Statistics 20.0 (SPSS Inc, Chicago, USA), and *P *< .05 was considered to be significant.

## 3 RESULTS

### 3.1 miR‐216a is up‐regulated in senescent endothelial cells

**Figure 1:**
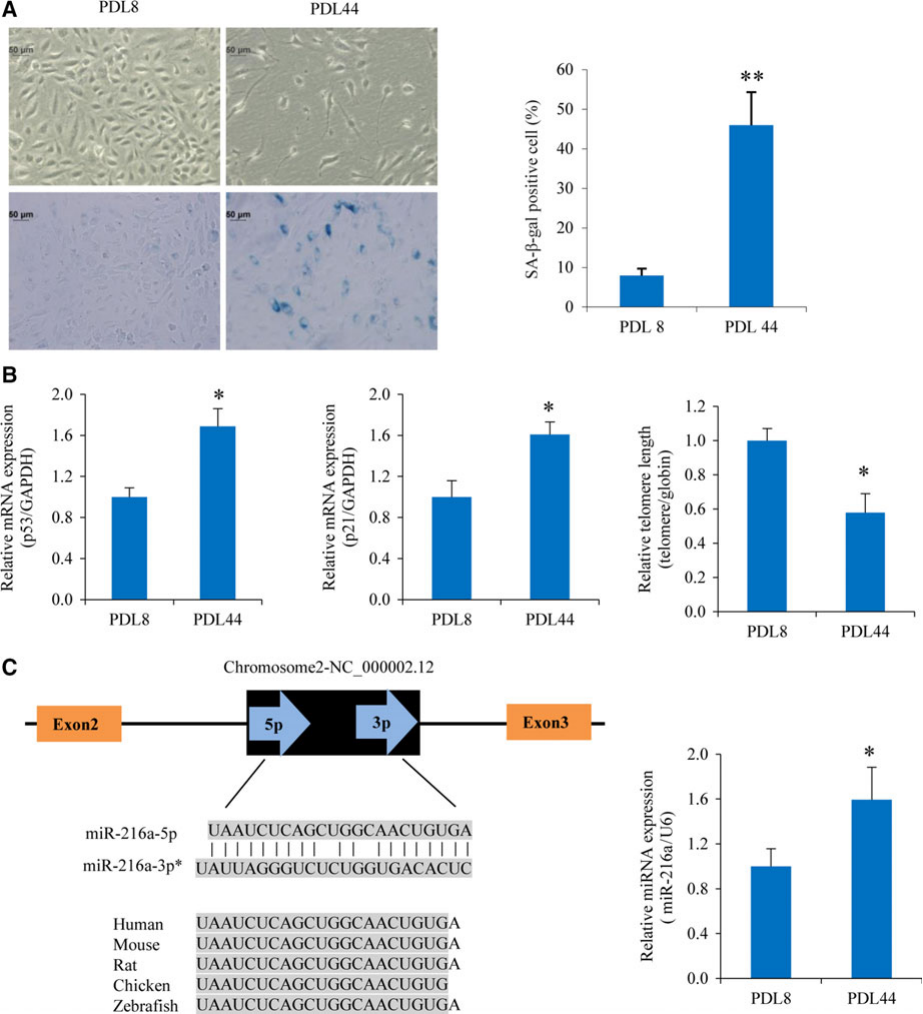
miR‐216a is up‐regulated in ageing endothelial cells. (A) Typical photos of staining for SA‐β‐gal of PDL8 and PDL44 HUVECs (n = 5). (B) The mRNA expression of senescence‐related genes p53, p21 and telomere length were analyzed by real‐time PCR (n = 3). (C) miR‐216a genomic organizations and conservation, and the expression level of miR‐216a during endothelial senescence (n = 3). Scale bars: 50 μm. *P < .05, **P < .01

The expression of miR‐216a during endothelial senescence was assessed in a replicative senescence model of HUVECs, and PDLs were calculated during cell passages. The results showed that ageing PDL44 HUVECs presented flattened and enlarged shape comparing to the round shape of young PDL8 HUVECs. The percentage of SA‐β‐gal positive‐staining cells was markedly increased by 4.7‐fold (*P *< .001) in senescent PDL44 HUVECs comparing with PDL8 HUVECs (Figure 1A). The expression levels of senescence‐related genes p53 and p21 were, respectively, up‐regulated by 69% (*P *= .003) and 61% (*P* = .001) in PDL44 HUVECs. In addition, telomere, the specialized DNA‐protein structure located at the end of chromosomes, is believed to be a marker for biological clock that determines cellular behaviour at the cellular level, with shorter telomere indicating cellular ageing. As expected, telomere length in PDL44 HUVECs was shorter by 42% (*P* = .01) (Figure 1B). PDL8 and PDL44 cells were identified as young and ageing endothelial cells, respectively.

miR‐216a, located at chromosome 2p16.1, was highly conservative in different species. The expression of miR‐216a was almost absent in young PDL8 and markedly up‐regulated by 59% in senescent PDL44 cells (*P* = .03) during endothelial ageing (Figure 1C). The miR‐216 family consists of two members, miR‐216a and miR‐216b. Compared with PDL8 cells, miR‐216b was not significantly increased in PDL44 cells (Figure S1).

To explore the expression of plasma miR‐216a under atherosclerotic stimuli during endothelial senescence, the young PDL8, middle PDL20 and senescent PDL44 lines were treated with ox‐LDL. The results showed that miR‐216a expression remained an increasing trend in response to ox‐LDL stimuli during the endothelial ageing process. However, only in old PDL44 cells, ox‐LDL stimuli significantly promoted miR‐216a expression by 65% (*P* = .04) compared to the ox‐LDL‐untreated group (Figure S2).

### 3.2 Overexpression of miR‐216a induces a premature senescent‐like phenotype in endothelial cells

**Figure 2:**
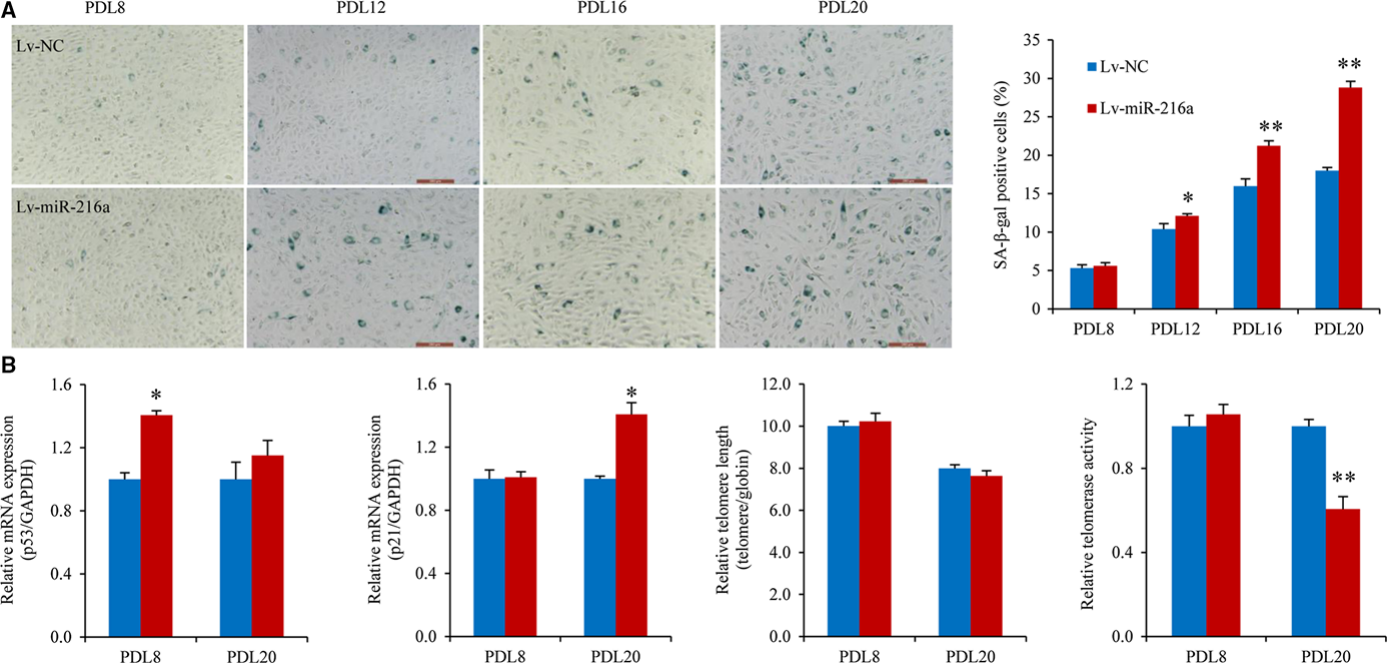
miR‐216a induces a premature senescent‐like phenotype in endothelial cells. (A) Photomicrographs of SA‐β‐gal staining of PDL8, PDL12, PDL16 and PDL20 stable line infected with miR‐216a or NC lentiviruses (n = 5). (B) The mRNA expression of p53 and p21, telomere length and telomerase activity in PDL8 and PDL20 stable line (n = 3). Scale bars: 200 μm. *P < .05, **P < .001

To investigate whether miR‐216a promoted the process of endothelial senescence, miR‐216a stable line was constructed by transfection with pre‐miR‐216a recombinant lentiviruses (Lv‐miR‐216a) in HUVECs and serially passaged. The data showed that miR‐216a significantly promoted the progression of cell senescence; with the increase in passage number, miR‐216a stable line showed a characteristic feature of growth arrest and larger cell morphology related to senescence (Figure 2A). Overexpression of miR‐216a increased the percentage of SA‐β‐gal‐positive cells by 20% (*P* = .05), 31% (*P* = .004), 61% (*P *= .001) in PDL12, PDL16 and PDL20 cells, separately (Figure 2A). The PDLs‐versus‐time growth curves showed that HUVECs with miR‐216a stable transfection (Lv‐miR‐216a) had a much slower growth rate than naturally passaged cells, and reached a significant growth arrest at about PDL20 which was similar as the senescent status of naturally passaged PDL44 (Figure S3). We therefore used PDL20 stable line to explore the effects of miR‐216a.

We further detected the expression of senescence‐related cyclins including p53 and p21, telomere length and telomerase activity. Stable overexpression of miR‐216a significantly up‐regulated p53 and p21 mRNA levels and down‐regulated telomerase activity in PDL8 and PDL20 stable lines, respectively. Telomere length remarkably shortened with the increase of PDLs, although no significant difference was observed between negative control and the stable miR‐216a lentiviral infection cells (Figure 2B).

### 3.3 miR‐216a promotes monocytes adhesion to endothelial cells monolayers

**Figure 3:**
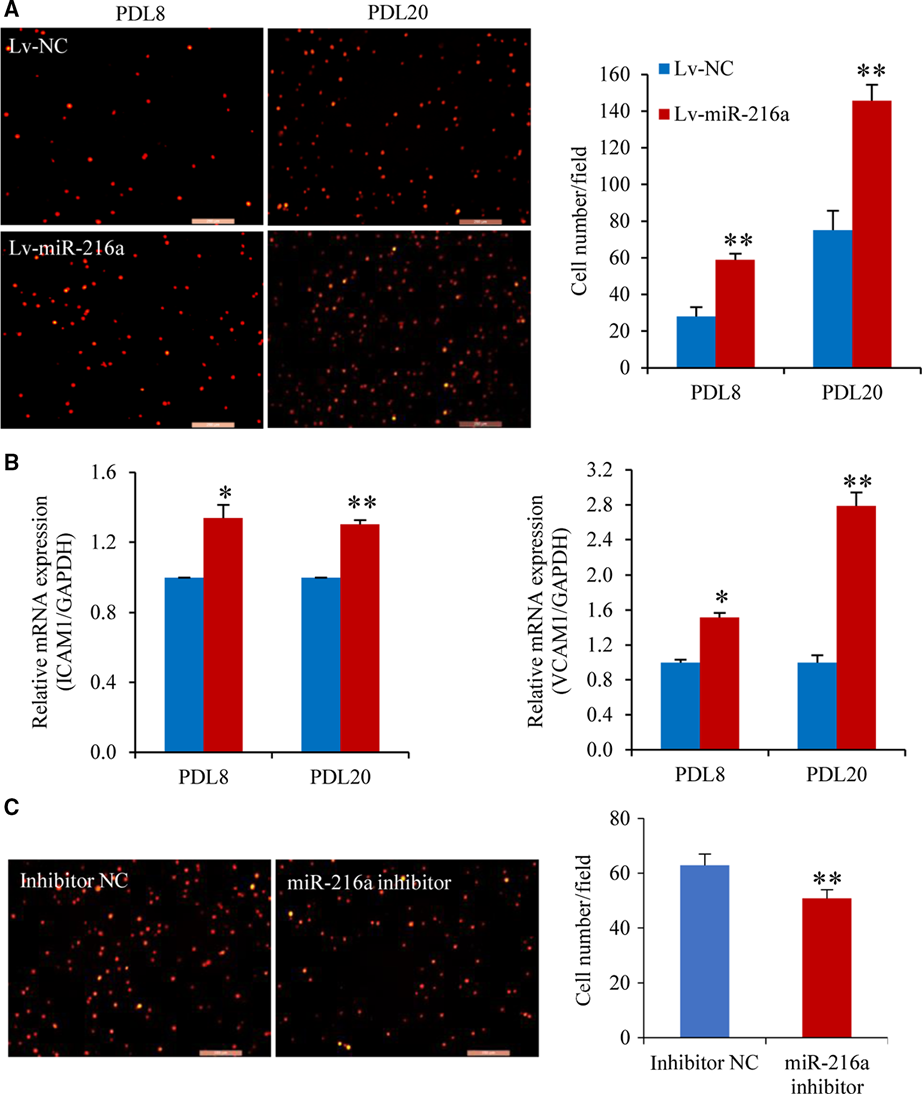
miR‐216a promotes monocytes adhesion to HUVECs monolayers. (A) Photo images of THP‐1 cells adhering to PDL8 and PDL20 cells with stable miR‐216a expression (n = 5). (B) The mRNA levels of adhesion molecules ICAM1 and VCAM1 in PDL8 and PDL20 stable line (n = 3). (C) The effects of antagomiR‐216a on the adhesion ability of THP‐1 cells to senescent PDL44 cells (n = 5). Scale bars: 200 μm. *P < .05, **P < .01

Because the PDL20 with miR‐216a stable transfection (Lv‐miR‐216a) showed an enlarged shape and slow growth rate which was similar as the senescent status of naturally passaged PDL44, we therefore used PDL20 stable line to explore the effects of miR216a on endothelial functions. The results showed that miR‐216a increased the adhesion ability of THP‐1 cells to HUVECs by 106% (*P* = .002) and 95% (*P* = .001) (Figure 3A) in PDL8 and PDL20 stable line of Lv‐miR216a, respectively. The expressions of adhesion molecules ICAM1 and VCAM1 were also remarkably increased by 34% and 52% in PDL8 Lv‐miR216a cells and were up‐regulated by 30% and 1.79‐fold in PDL20 Lv‐miR216a cells, respectively (Figure 3B).

On the other hand, for the experiments of miR‐216a inhibitor, the naturally senescent PDL44 line was used and transiently transfected by antagomiR‐216a to explore the changes of endothelial functions after the inhibition of endogenous miR‐216a. Once miR‐216a function was inhibited, the endothelial adhesion ability of to THP‐1 cells was decreased by 19% (*P* = .01) (Figure 3C).

### 3.4 miR‐216a inhibits proliferation and migration during endothelial cells growth

**Figure 4:**
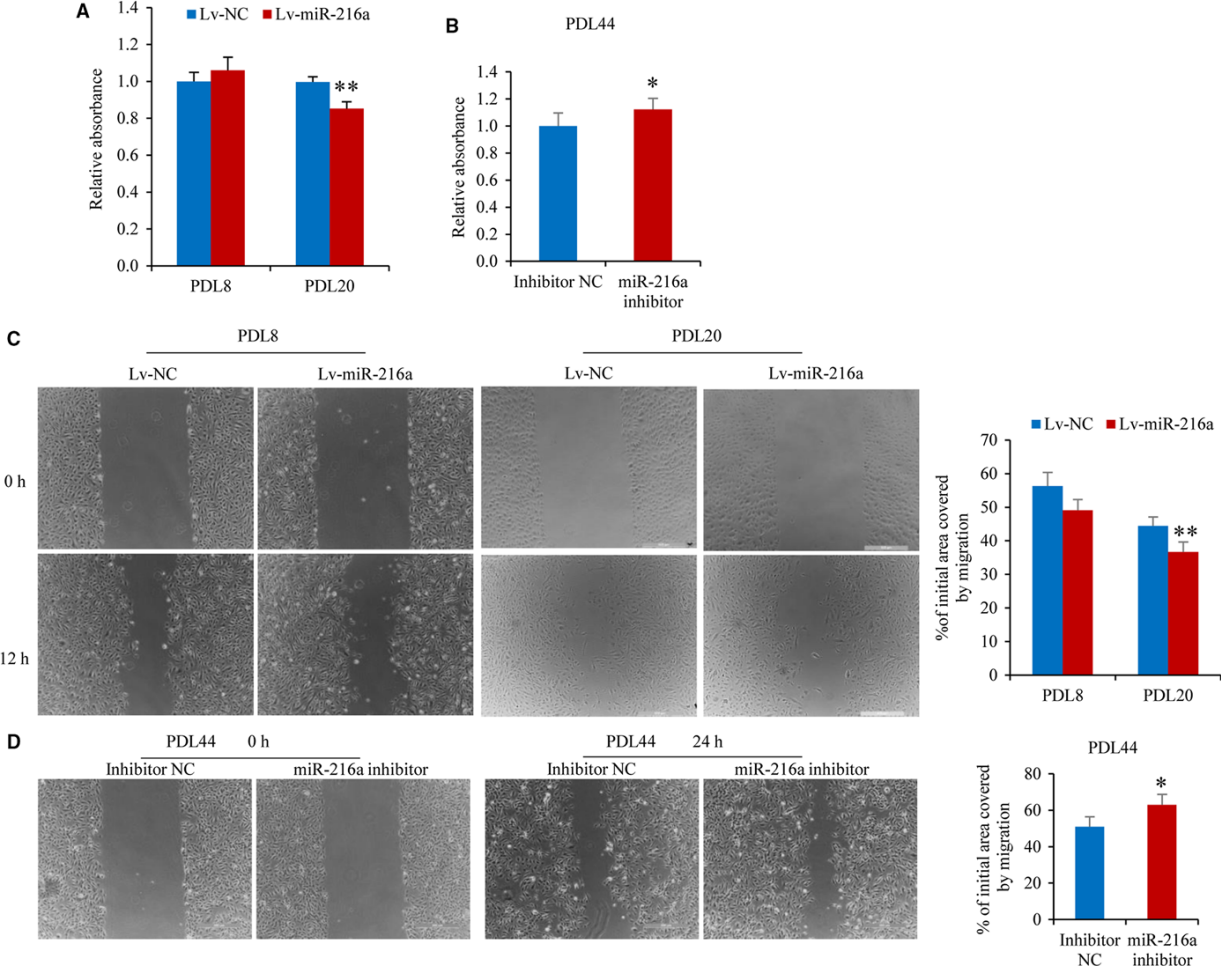
miR‐216a inhibits proliferation and migration during endothelial cells growth. (A) The proliferation activity of PDL8 and PDL20 stable line was performed by MTS assay (n = 8). (B) The effect of miR‐216a inhibition on proliferation activity of senescent PDL44 HUVECs (n = 8). (C) Migration ability of PDL8 and PDL20 stable line was assessed by scratch assay after a scratch in confluent monolayer of cells for 12 hours (n = 5). (D) Migration ability of senescent PDL44 HUVECs with transfection of miR‐216a inhibitor was assessed after a scratch for 24 hours (n = 5). Scale bars: 500 μm. *P < .05, **P < .01

The MTS assay, scratch test and tube formation assay were performed to assess the effects of miR‐216a on endothelial cells proliferation, migration and angiogenesis function, respectively. miR‐216a overexpression was not found to change the cells proliferation activity and wound healing ability at the beginning of infection with miR‐216a in PDL8 cells. After infection at about 15 days, stable overexpression of miR‐216a suppressed cells proliferation by 15% (*P *< .001) and migration ability by 16% (*P *< .001) in senescent PDL20 stable line, respectively (Figure 4A and C).

Similarly, antagomiR‐216a was transiently transfected into naturally senescent PDL44 line to investigate the effect of miR‐216a on proliferation and migration. Once miR‐216a function was inhibited, the endothelial proliferative ability was increased by 12% (*P *= .02) and migration ability was increased by 24% (*P *= .03), respectively (Figure 4B and D). Additionally, miR‐216a had no significant effect on angiogenic activity of endothelial cells in both young PDL8 and senescent PDL20 stable line of Lv‐miR‐216a (Figure S4).

### 3.5 Smad3 is a direct target of miR‐216a

**Figure 5:**
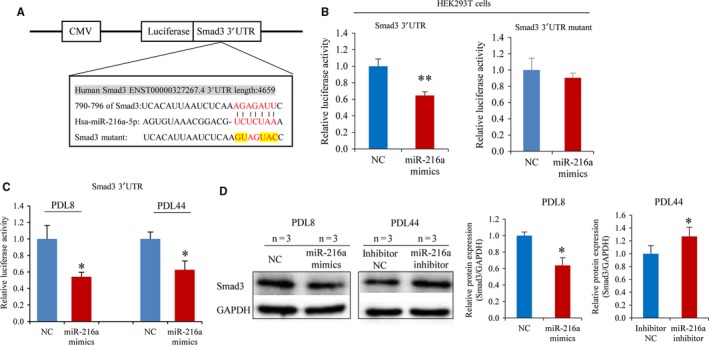
Smad3 is a direct target of miR‐216a. (A) The potential binding sites of miR‐216a and the mutations in miR‐216a binding sites within Smad3 3′UTR. (B) The effects of miR‐216a on luciferase activity of HEK293T cells transfected with Smad3 3′UTR or 3′UTR mutant luciferase reporter vector (n = 8). (C) The effects of miR‐216a on luciferase activity of PDL8 and PDL44 HUVECs transfected with Smad3 3′UTR luciferase reporter vector (n = 8). (D) The protein levels of Smad3 in young and senescent HUVECs transfected with miR‐216a mimics and miR‐216a inhibitor, respectively (n = 3). *P < .05, **P < .01

From the above results, we confirmed that the overexpression of miR‐216a indeed induced endothelial senescence and promoted endothelial–monocytes interaction. Computational miRNA target analysis by TargetScan Release 7.1 from miRNA databases indicates that Smad3 is a potential target of miR‐216a and contains a candidate miR‐216a binding site between 790 and 796 bps in the Smad3 3′UTR (Figure 5A). Next, the luciferase reporter vectors of Smad3 3′UTR and 3′UTR mutant were, respectively, constructed and used to examine the effect of miR‐216a on Smad3 mRNA level. Co‐transfection of miR‐216a mimics with Smad3 3′UTR reporter in HEK293T cells inhibited luciferase activity by 40% (*P *< .001), compared with that transfected with negative control. As expected, the luciferase reporter carrying mutations in the miR‐26a binding site was unresponsive to miR‐216a (Figure 5B).

To verify the regulatory role of miR‐216a on Smad3 3′UTR in young and old HUVECs, PDL8 and PDL44 HUVECs were co‐transfected with miR‐216a and Smad3 3′UTR luciferase reporter. The results showed that miR‐216a inhibited luciferase activity by 46% (*P* = .03) and by 37% (*P* = .02) in PDL8 and PDL44 HUVECs, respectively (Figure 5C).

Furthermore, young PDL8 and senescent PDL44 HUVECs were separately transfected with miR‐216a mimics and miR‐216a inhibitor. After 48 hours, Smad3 protein levels were significantly decreased in PDL8 HUVECs by miR‐216a mimics compared to negative control. On the contrary, transfection with an equal amount of miR‐216a inhibitor increased Smad3 protein expression in PDL44 cells (Figure 5D).

### 3.6 miR‐216a promotes endothelial inflammation through Smad3/IκBα pathway

**Figure 6:**
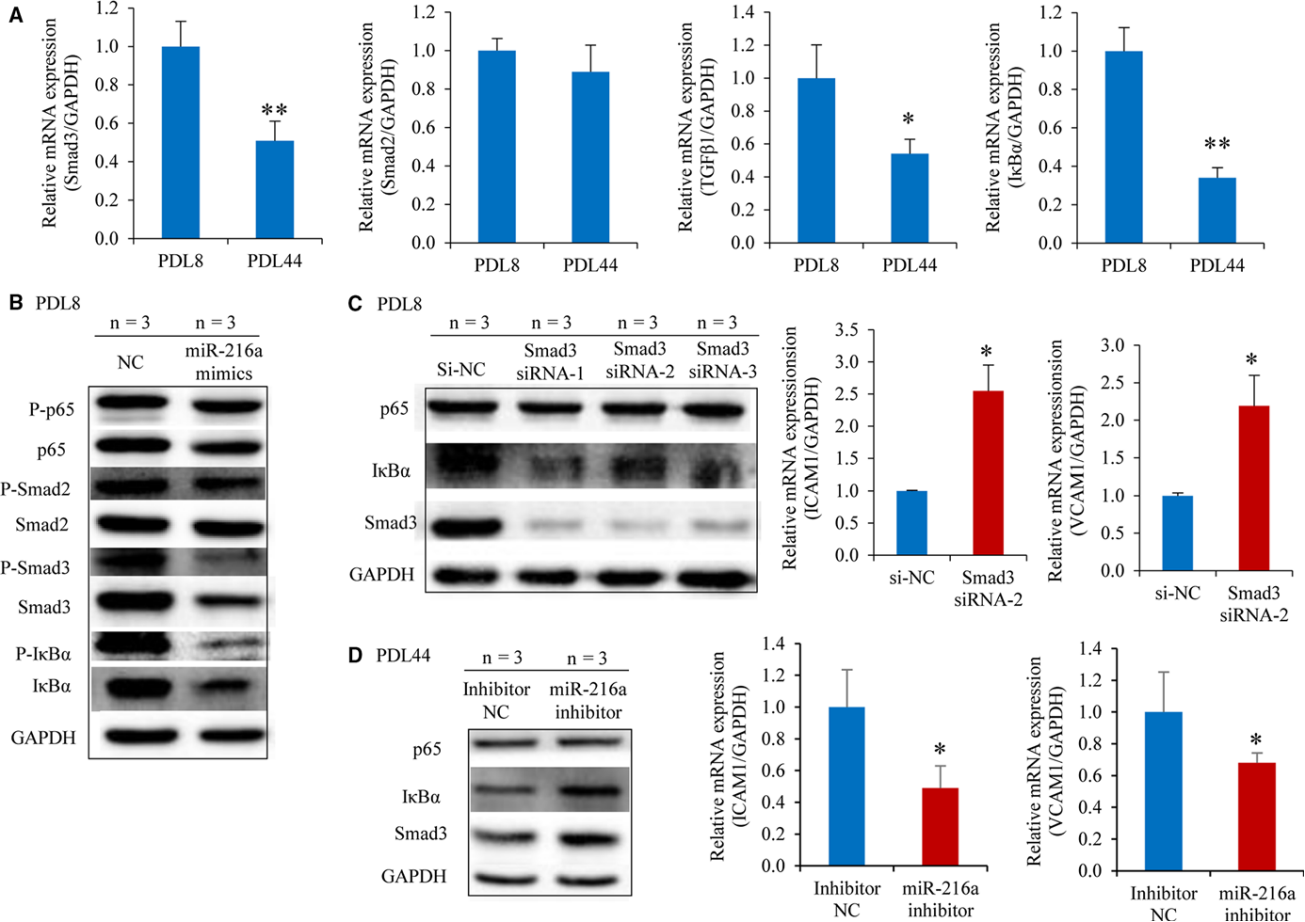
miR‐216a promotes endothelial inflammation through Smad3/IκBα pathway. (A) The mRNA expression of Smad3, TGFβ1, Smad2 and IκBα during HUVECs senescence. (B) The protein levels of Smad3, Smad2, p65, IκBα and their phosphorylation levels in young PDL8 cells with miR‐216a mimics transfection. (C) The protein levels of Smad3, p65 and IκBα and the mRNA expression of ICAM1 and VCAM1 by silencing Smad3 in PDL8 cells. (D) The Smad3 and IκBα protein levels and ICAM1 and VCAM1 mRNA expression after inhibition of miR‐216a in senescent PDL44 cells. n = 3 for each group, *P < .05, **P < .01

Smad3 is an important member of TGFβ1 pathway family and mediates the anti‐inflammatory effects of TGFβ1. To investigate whether miR‐216a regulates endothelial inflammation by targeting Smad3, the expression of Smad3 and its pathway genes including TGFβ1, Smad2 and downstream IκBα was examined in PDL8 and PDL44 HUVECs. The results showed that mRNA expression levels of TGFβ1, Smad3 and IκBα were markedly decreased in senescent PDL44 as compared to young PDL8 HUVECs; moreover, this decrease was negatively correlated with natural miR‐216a level. There was no correlation between Smad2 mRNA expression and miR‐216a level (Figure 6A).

In PDL8 cells, overexpression of miR‐216a resulted in marked degradation of Smad3 as well as IκBα, but no significant change on Smad2, p65 protein levels and their phosphorylation (Figure 6B). Next, to confirm the negative regulation of miR‐216a on IκBα, Smad3 siRNAs and miR‐216a inhibitor were transfected to PDL8 and PDL44 cells, respectively. As expected, IκBα expression was diminished by silencing Smad3, and consequently, the mRNA expression levels of downstream adhesion molecules ICAM1 and VCAM1 were up‐regulated (Figure 6C). On the contrary, once endogenous miR‐216a was inhibited, Smad3 and IκBα expression were up‐regulated and ICAM1 and VCAM1 expression were down‐regulated (Figure 6D). All these results supported the role of miR‐216a as a natural inhibitor of Smad3 in promoting endothelial inflammation *via* Smad3/IκBα pathway.

### 3.7 Plasma miR‐216a level is elevated in old patients with coronary artery diseases

**Figure 7:**
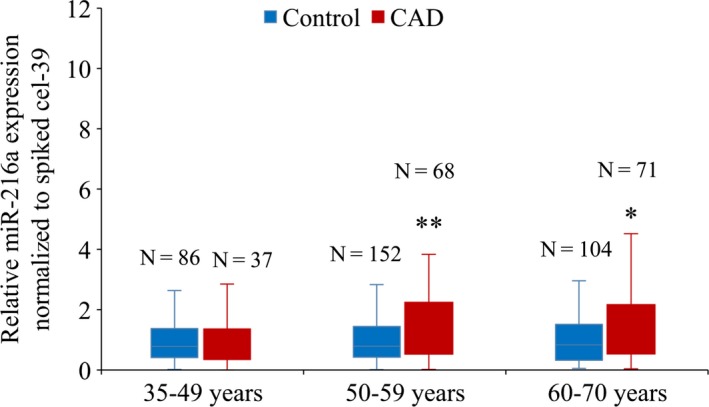
Plasma miR‐216a level is elevated in elderly patients with coronary artery diseases. CAD patients (n = 176) and a corresponding control group (n = 342) are divided into three age groups (age 35‐49 years, 50‐59 years, >60 years). No marked difference was observed between young CAD patients (0.84, IQR, 0.43‐1.75) and controls (0.82, IQR, 0.42‐1.65) (*P *= .80) in age 35‐49 years group. Plasma miR‐216a level was significantly higher in elderly CAD patients (1.55, IQR, 0.73‐4.00) compared with controls (0.80, IQR, 0.43‐1.60) in age 50‐59 years group (*P *= .001). Plasma miR‐216a level was obviously elevated in aged CAD patients (1.38, IQR, 0.58‐2.68) compared with controls (0.88, IQR, 0.36‐1.57) in age >60 years group (*P *= .004). *P < .05, **P < .01

The regulatory effects of miR‐216a on endothelial senescence and dysfunction suggest an important role of miR‐216a in the pathogenesis of age‐associated vascular diseases. To assess the association between miR‐216a and risk of CAD, particularly in those ageing individuals, plasma miR‐216a levels were examined in 176 CAD patients and 342 age, gender‐matched healthy controls. The clinical characteristics of patients and controls were described in Table S2. Plasma miR‐216a level was significantly elevated in CAD patients (1.11, IQR, 0.49‐2.02) compared with in healthy controls (0.79, IQR, 0.39‐1.47) (*P *= .002). After stratified by age (35‐49 years, 50‐59 years and >60 years), miR‐216a plasma level was significantly higher in elderly CAD patients (>50 years) than those healthy controls (>50 years), whereas no markedly difference was found between young CAD patients and controls (<50 years) (*P *= .80) (Figure 7).

After adjustment for conventional vascular risk factors including age, gender, body mass index, cigarette smoking, alcohol consumption, blood pressure, fasting glucose and lipid profiles, plasma miR‐216a was associated with a 31% increased risk for CAD (OR = 1.31, 95% CI 1.03‐1.66; *P *= .03) in >50 years of age group, while no association was observed in young CAD patients (<50 years of age group) (OR = 0.83, 95% CI 0.42‐1.64; *P *= .60).

## 4 DISCUSSION

Vascular endothelial cells senescence is one of major risk factors for atherosclerosis and cardiovascular diseases. Emerging evidence indicate that miRNAs represent a common molecular mechanism that underlies the cell senescence. In the current study, we provided evidence for the first time that the age‐progressive increase in miR‐216a accelerated endothelial senescence and promoted endothelial inflammation as an endogenous inhibitor of Smad3/IκBα signalling pathway. Consistently, our analysis showed that plasma miR‐216a level significantly elevated in CAD patients compared with healthy controls, particularly in old CAD patients (>50 years). These findings indicated that miR‐216a might serve as a novel biomarker and therapeutic target for endothelial dysfunction and related atherosclerotic diseases in aged individuals.

miR‐216 has been previously shown a significant differentially expression when compared the miRNA profile obtained in senescent human endothelial cells *versus* young cells,^6^ but its role in endothelial ageing remained unclear. Our data verified that miR‐216a expression was absent in young endothelial cells and progressively increased in ageing cells during repeated rounds of cellular replication. Stable overexpression of miR‐216a induced a premature senescent‐like phenotype in endothelial cells with impairment of proliferation and migration ability; moreover, miR‐216a overexpression increased adhesion ability to monocytes both in young and senescent cells, while inhibition of miR‐216a decreased, in part, adhesion ability in senecent cells. Furthermore, we showed that the mechanism linking miR‐216a to endothelial senescence and inflammation was involved in Smad3/IκBα pathway.

Smad3/IκBα signalling pathway is the main downstream mediator of TGFβ1 and responsible for its anti‐inflammatory effect.^11^ TGFβ1 has been reported to express in vascular wall, including endothelial cells, vascular smooth muscle cells, monocytes/macrophages, regulatory T cells and myofibroblasts.^16^ Expression of TGFβ1 and receptors is increased in human atherosclerotic plaque lesion, especially in fibro‐proliferative regions, compared with non‐atherosclerotic regions.^17^, ^18^ TGFβ1 can stimulate endothelial migration, proliferation and angiogenesis at low concentrations, but inhibit these functions at higher concentrations, which associated with increased extracellular matrix.^19^ In this study, the TGFβ1 expression in senescent PDL44 HUVECs was significantly decreased compared with that in young PDL8 cell line.

In response to inflammatory stimuli, vascular endothelial cells express a number of adhesion molecules that mediate early leukocyte attachment and atherosclerosis progression, particularly, such as ICAM1, VCAM1 and E‐selectin. Here, in our study, Smad3 was demonstrated as a target of miR‐216a; Smad3 and IκBα expression significantly decreased in ageing endothelial cells, negatively correlated with miR‐216a. We also found that miR‐216a inhibited Smad3 protein expression and mediated downstream IκBα degradation, and thus activated NF‐κB responsive genes such as ICAM1 and VCAM1 in the endothelial cells, which thereby promoted adhesion ability of endothelial cells to monocytes. Furthermore, siRNA could “phenocopy” the effects of miR‐216a on Smad3, IκBα, and adhesion molecules ICAM1 and VCAM1; on the contrary, once endogenous miR‐216a was reduced by its synthetic inhibitor, the Smad3/IκBα expression was rescued. These results supported the concept that miR‐216a can promote endothelial inflammation by serving as an endogenous inhibitor for the Smad3/IκBα pathway during endothelial cell growth and ageing.

On a cellular level, ageing is usually defined as replicative senescence which occurs *in vitro* after a certain number of cell cycles. Our data showed that miR‐216a expression was absent in young endothelial cells and progressively increased in ageing cells during repeated rounds of cellular replication in the cultured HUVECs *in vitro*. On the other hand, in the healthy aged individuals (>50 or >60 years), plasma miR‐216a level showed no significant changes compared to young controls, whereas coronary arterial disease was found to promote expression of miR‐216a in aged patients compared to controls. This may be partially explained by the complexity of ageing mechanism in response to the intracellular and extracellular stressors.

It is well‐recognized that the burden of chronic inflammation and oxidative stress can accumulate during the individual's life course and contribute to the progression of atherosclerosis and age‐dependent vascular diseases. Consistent with this concept, when the young PDL8, middle PDL20 and senescent PDL44 HUVECs were treated with ox‐LDL, a crucial atherogenic factor, we found that miR‐216a was significantly up‐regulated by ox‐LDL in old endothelial cells, but not in young cells, suggesting a relevant influence of inflammatory environment on the expression of plasma miR‐216a. Healthy persons might have lower levels of circulating pro‐inflammatory cytokines and pro‐atherogenic factors and maintain endothelial functional integrality, whereas in patients with coronary heart disease, endothelial cells may exhibit more serious dysfunction and inflammatory status in old age which thus result in a remarkably increased plasma miR‐216a in old patients. Further experimental evidence *in vivo* and a larger cohort will help confirm a potential role of miR‐216a in atherosclerosis under the inflammatory environment.

miR‐216a has recently been reported to be related to the initiation and progression of atherosclerosis. For example, in THP‐1 macrophages‐derived foam cells, miR‐216a regulated ATP‐binding cassette transporter A1 (ABCA1)‐mediated cholesterol efflux by targeting cystathionine γ‐lyase.^20^ In endothelial cells, miR‐216a controlled ox‐LDL‐induced autophagy by suppressing intracellular levels of Beclin 1, and inhibition of miR‐216a can rescue the impairment of autophagy during endothelial ageing.^21^ However, the causal link between miR‐216a and endothelial senescence was not studied in these reports. Here, we first clarified that miR‐216a induced endothelial senescence and promoted endothelial inflammation *via* Smad3/IκBα pathway, which thus extended the knowledge on miR‐216a.

In the current study, our results found that the presence of increased SA‐β‐gal activity was later than the elevation of p53 expression in PDL8 line after miR‐216a transfection for 3 days. p53 is well‐recognized to play a key role in cell‐cycle control, DNA repair and cellular stress responses, and recent studies has reported that p53 activation of p53 can regulate apoptosis, cellular senescence and organismal ageing.^22^ SA‐β‐gal is an extensively utilized marker for cell senescence, and the β‐galactosidase activity can be detected at pH 6.0 in replicative or induced senescence cells but undetectable from proliferating cells.^23^ Levels of p53 often gradually increase during the process of replicative senescence, whereas the presence of SA‐β‐gal marker can distinguish senescent cells during cell passages independent of DNA synthesis.^23^ Thus, a time‐lag may exist between the time‐point of increased p53 levels and the presence of SA‐β‐gal activity. For example, it has been reported that exogenous IGF‐binding protein 5 (IGFBP‐5) markedly increased p53 expression after 90 minutes in HUVECs, while SA‐β‐gal activity was found to be elevated after prolonged treatment IGFBP‐5 for 6 days.^24^

miR‐216a is expressed in several vascular cells, including monocytes/macrophages,^20^ vascular smooth muscle cells and senescent endothelial cells.^21^ Circulating endothelial progenitor cells (EPCs) can modulate the repair and regeneration of the endothelium associated with vascular ageing, atherosclerosis and CAD. It has been reported that elderly healthy subjects had lower circulating EPCs with impaired function and increased senescence when compared to young people.^25^ Circulating EPCs obtained from CAD patients, compared with from age‐matched healthy subjects, showed shorter telomere length and lower telomerase activity.^26^ These emerging evidence supported that the decreased number and increased senescence of circulating EPCs in older CAD patients contribute to vascular endothelial dysfunction and ageing. Further comparison of miR‐216a expression in plasma and circulating angiogenic cells with senescent phenotype between older and younger CAD patients, such as EPCs, would help clarify the underlying mechanisms of miR‐216a in vascular repair and ageing related to atherosclerotic diseases.

In summary, we provided evidence for the first time that the age‐progressive increase in miR‐216a triggers endothelial senescence and promotes the endothelial inflammation by targeting Smad3 and leading to NF‐κB signalling activation. Moreover, in aged CAD patients, miR‐216a plasma level was significantly higher than in healthy controls. This extends the knowledge of mechanisms involved in both the functions of miRNAs and their application serving as new potential biomarkers and therapeutic targets for endothelial dysfunction and related atherosclerotic diseases in aged individuals.

## CONFLICTS OF INTEREST

We have no conflicts of interest to disclose.
