# Supplementary material for: MicroRNA‐216a induces endothelial senescence and inflammation via Smad3/IκBα pathway
Source: J Cell Mol Med. 2018 Mar 7;22(5):2739–49. doi: 10.1111/jcmm.13567 (PMC5908109; doi:10.1111/jcmm.13567)
Supplement: Supplementary file 1 [file JCMM-22-2739-s001.doc]

**Supplementary Materials**

**Title:** MicroRNA-216a induces endothelial senescence and inflammation *via* Smad3/IκBα pathway

**Methods**

**Cell culture**

Primary human umbilical vein endothelial cells (HUVECs) were cultured using endothelial cell medium (ECM) (ScienCell, San Diego, CA, USA) added with 5% fetal bovine serum (FBS) and population-doubling levels (PDLs) during passages were calculated [1]. In brief, the number of PDLs was calculated by the equation: PDL= log_2_(Ch/Cs), in which Ch is defined as the number of viable cells at harvest and Cs as the number of cells seeded. A series of HUVECs from PDL4 to PDL40 were frozen for later experiments. When the PDL44 line was needed, one tube of PDL40 line was thawed and passaged to PDL44 to explore the endothelial functions, including the wound healing assay as well as proliferation, migration, and adhesion ability assays. It is possible that the time after cryopreservation, the minor difference of medium batch and especially the density of cells would affect the senescent cell morphology. Before the experiments, the senescent status of PDL44 line was assessed. PDL8 and PDL44 cells were identified as young and senescent HUVECs, respectively.

In addition, to explore the expression of miR-216a under atherosclerotic stimuli during endothelial senescence, a series of PDL8, PDL20 and PDL44 HUVECs were incubated with 25 μg/ml ox-LDL (Yiyuan Biotech, Guangzhou, China) for 48 hours at 37℃.

**Senescence-associated *β*-galactosidase staining**

HUVECs senescence were identified by *in situ* staining for senescence-related *β*-galactosidase (SA-*β*-gal) with staining kit (Beyotime Biotechnology, Shanghai, China). Briefly, cells were fixed at room temperature for 10 minutes in 4% paraformaldehyde solution. After washed with PBS buffer, cells were incubated with fresh *β*-galactosidase staining solution (pH 6.0) for 16 hours at 37°C without CO_2_. Photos of the staining were acquired in more than 5 different microscopic fields per sample and the percentages of SA-*β*-gal positive cells were calculated by counting 5 fields.

**Cell adhesion assay**

To assess the effects of miR-216a on endothelial adhesive capacity to monocytes, cell adhesion assay was performed. PDL8 and PDL20 stable line were respectively seeded in a 24-well plate in ECM complete medium, grown to 90% confluency. Additionally, PDL44 cells was plated and transfected with miR-216a inhibitor for 48 hours. The THP-1 cells (China Infrastructure of Cell Line Resources, Beijing, China) were washed twice with serum-free RPMI-1640 medium, then resuspended at a density of 1×10^6^ cells/ml. The 1 ml THP-1 cells were labeled with 5 μl CellTracker^TM^ CM-Dil (Invitrogen, Carlsbad, CA, USA) for 30 minutes at 37℃, washed twice with PBS buffer, and then were resuspended in the RPMI-1640 medium at 1×10^6^ cells/ml. The labeled cells were added to each well (200 μl/well) containing HUVECs and kept in an incubator containing 5% CO_2_ at 37℃ for 30 minutes. The non-adherent THP-1 cells were removed by PBS, and the adherent cells were counted in 5 random fields.

**Cell proliferation assay**

To assess the role of miR-216a during endothelial cells growth, the MTS ([3-(4,5-dimethylthiazol-2-yl)-5-(3-carboxymethoxyphenyl)-2-(4-sulfophenyl)-2H-tetrazolium]) assay was performed in PDL8 and PDL20 stable line, respectively. Cells were seeded at a density of 1×10^4^ cells/well onto 96-well plates for overnight growth. CellTiter 96® AQ_ueous_ One Solution Cell Proliferation Assay Kit (Promega, Madison, MI, USA) was applied and the absorbance of samples was detected at a dual wavelength of 490 nm and 630 nm using the LB960 microplate reader (Berthold, Bad Wildbad, Germany).

**Cell migration assay**

The scratch test was conducted to investigate the role of miR-216a on endothelial cells migration and wound healing. Briefly, the PDL8 and PDL20 transfected cells were respectively seeded onto 12-well plates and cultured to 95% confluence. Cell monolayer was scratched with 200-μl tip to create wound gaps, washed twice with serum-free ECM, and cultured in ECM containing 1.25% FBS for 12 hours. The wound gaps were photographed at 0 hours and 12 hours with a light microscope DMI-4000B (Leica, Wetzlar, Germany), respectively. Wound healing rate was calculated according to the equation: % wound healing = [(Area of original wound - Area of wound after healing)/Area of original wound] × 100%. Each wound was analyzed in 5 different areas.

**Cell angiogenesis assay**

The tube formation assay was performed to assess the effects of miR-216a on angiogenic activity in PDL8 and PDL20 transfected cells, respectively. Briefly, cells were plated with a density of 5×10^4^ cells/well onto 48-well plate pre-coated with 100 µl/well growth factor-reduced Matrigel (BD Biosciences, Franklin Lakes, NJ, USA). After incubation with ECM containing 1.25% FBS for 6 hours, tube formation was photographed in 5 random fields by Leica DMI-4000B microscope. Tube formation was identified as a tube-like structure with a length four times its width, and the cumulative mean tube lengths per field of view were quantified.

**Telomerase enzyme activity and telomere length measurement**

To assess the telomerase activity during endothelial senescence, real-time PCR was performed [2]. A total of 1×10^5^ transfected cells were resuspended in 100 μl CHAPS buffer (Roche, Mannheim, Germany) and incubated for 30 minutes on ice. After centrifugation for 30 minutes at 16000 g at 4°C, cell lysate supernatant was used as template for amplification. Real-time PCR was performed with 0.5 U Hotstar DNA polymerase (Takara, Dalian, China), 200 nM TS primers, 100 nM Cxa primers, 0.4×SYBR-Green, 10×buffer and dNTP on the ABI 7500 System (Applied Biosystems, Foster City, CA, USA). The primers were as the following: TS primers 5’-AATCCGTCGAGAACAGTT-3’, Cxa primers 5’-GTGTAACCCTAACCCTAA CCC-3’. The reaction program was 37℃ for 30 minutes, 95℃ for 5 minutes, followed by 40 cycles of 95℃ for 15 seconds, 50℃ for 30 seconds, 72℃ for 30 seconds.

To measure the telomere length between young and aging endothelial cells, genomic DNA was extracted from cells by MiniBEST Universal Genomic DNA Extraction Kit (Takara, Dalian, China). Relative mean telomere length in endothelial cells was determined by a quantitative real-time PCR method which compares telomere repeat copy number (T) to single-copy gene copy number β-globin (S) (T/S ratio) [3]. In brief, real-time PCR was performed with Hotstar DNA polymerase on the ABI 7500 System. A standard curve was examined by using serially diluted reference DNA from HEK293T cells (100 ng; 2-fold dilution; seven points) was run with good linearity (R^2^ > 0.97) for both the telomere and the β-globin real-time PCRs. The primers were as the following: for telomere measurement, forward 5’-CGGTTTGTTTGGGTTTGGGTTTGGGTTTGGGTTTGGGTT-3’and reverse 5’-GGCTTGCCTTACCCTTACCCTTACCCTTACCCTTACCCT-3’ were used; for β-globin measurement, forward 5’-GCTTCTGACACAACTGTGTTCACTAGC-3’ and reverse 5’-CACCAACTTCATCCACGTTCACC-3’ were used.

**Plasmid construction and luciferase reporter assay**

A total of 2,002 base pairs (bps) sequence within Smad3 3’UTR was PCR amplified with primers: forward 5' CTCAACGCGTGCGTCTGCTCTGGTGGCT 3', reverse 5' GGCGCAAGCTTCACCTGGAGTAAGACACGACTTC 3', which contains a miR-216a consensus response element located between 790 and 796 bps of sequence ENST00000327367.4. The amplicon was cloned into the downstream of ﬁreﬂy luciferase gene of the pMIR-REPORT^TM^ Luciferase plasmid (Ambion, Austin, TX, USA). The regulatory role of miR-216a on Smad3 mRNA level were assessed using luciferase reporter assay in HEK293T cells (China Infrastructure of Cell Line Resources, Beijing, China). Cells were co-transfected with 100 ng Smad3 3’UTR plasmid in the 96-well plate and miR-216a mimics or negative control (NC) at the concentration of 50 nM (n=8 per group) using Lipofectamine 3000. After transfection for 2 days, the luciferase activities of ﬁreﬂy and Renilla were datected by the Dual-Luciferase Reporter Assay System (Promega, Madison, WI, USA) using an UniCel DxC 800 Synchron Analyzer (Backman, CA, USA). Renilla luciferase activity was used to normalize fireﬂy luciferase activity.

**Western blot analysis**

The effects of miR-216a on Smad2, Smad3, p65 and NF-κB inhibitor alpha (IκBα) protein levels were determined by western blot assay. HUVECs were harvested and proteins extracts were isolated with RIPA lysis buffer (Beyotime Biotechnology, Shanghai, China) containing protease inhibitor (Roche, Mannheim, Germany) and their concentrations were determined by the Pierce BCA Protein Assay Kit (Invitrogen, Carlsbad, CA, USA). Each sample protein was loaded with 50 μg and electrophoresed on 10% SDS-PAGE gels, then transferred to nitrocellulose membranes (Millipore, MA, USA). After blocking with 5% BSA, the membranes were incubated by primary and secondary antibodies. Primary antibodies were rabbit polyclonal anti-Smad2, anti-Smad3, anti-p65, mouse monoclonal anti-IκBα, and their phosphorylated antibodies (Cell Signaling Technology, Danvers, MA, USA). Rabbit polyclonal anti-GAPDH (Cell Signaling Technology, Danvers, MA, USA) was applied as an internal reference. Correspondingly, secondary antibodies were anti-rabbit (1:5000) or anti-mouse (1:5000) antibodies labeled with horseradish peroxidase. Bands were visualized with FluorChem R, M and E Systems (ProteinSimple, CA, USA) and quantified with AlphaView Software.

**References**

1. **Menghini R, Casagrande V, Cardellini M*, et al.*** MicroRNA 217 modulates endothelial cell senescence via silent information regulator 1. *Circulation*. 2009; 120: 1524-32.

2. **Voglauer R, Grillari J, Fortschegger K*, et al.*** Establishment of human fibroma cell lines from a MEN1 patient by introduction of either hTERT or SV40 early region. *Int J Oncol*. 2005; 26: 961-70.

3. **Cawthon RM.** Telomere measurement by quantitative PCR. *Nucleic Acids Res*. 2002; 30: e47.

**Table S1** The primers for real-time PCR

|  | **Gene** |  | **Sequence (5' −> 3')** | |  |
| --- | --- | --- | --- | --- | --- |
| p53 | | forward | | CGTGTGGAGTATTTGGATGACAGA | |
|  |  | reverse | | GGAGTCTTCCAGTGTGATGATGGT | |
| p21 | | forward | | GAGCAGGCTGAAGGGTCCCCAGGT | |
|  |  | reverse | | GCTTCCTGTGGGCGGATTAGGGCT | |
| ICAM1 | | forward | | TCTGTGTCCCCCCTCAAAAGTC | |
|  |  | reverse | | GGGTCTCTATGCCCAACAA | |
| VCAM1 | | forward | | GATACAACCGTCTTGGTCAGCCC | |
|  |  | reverse | | CGCATCCTTCAACTGGCCTT | |
| TGFβ1 | | forward | | TACCTGAACCCGTGTTGCT | |
|  |  | reverse | | CAGTGTGTTATCCCTGCTGTC | |
| Smad2 | | forward | | GGATTTACAGCCAGTTAC | |
|  |  | reverse | | AAAGCCATCTACAGTGAG | |
| Smad3 | | forward | | CGTGCGGCTCTACTACATC | |
|  |  | reverse | | ACATTCGGGTCAACTGGT | |
| IκBα | | forward | | CACCAACCAGCCAGAAAT | |
|  |  | reverse | | ATCAGCACCCAAGGACAC | |
| GAPDH | | forward | | GAAGGTGAAGGTCGGAGTCA | |
|  |  | reverse | | GGAAGATGGTGATGGGATTTC | |

**Table S2** Clinical characteristics of patients with coronary artery diseases and controls in three age groups

| Characteristics | | Age 35-49 years (n=123) | | |  | Age 50-59 years (n=220) | | |  | Age >60 years (n=175) | | |
| --- | --- | --- | --- | --- | --- | --- | --- | --- | --- | --- | --- | --- |
|  | Controls  (n=86) | | Cases  (n=37) | *P* |  | Controls  (n=152) | Cases  (n=68) | *P* |  | Controls  (n=104) | Cases  (n=71) | *P* |
| Age, years | | 45.2 ± 3.0 | 45.0 ± 3.0 | 0.69 |  | 54.3 ± 3.0 | 54.4 ± 2.8 | 0.77 |  | 63.9 ± 3.2 | 64.1 ± 2.7 | 0.66 |
| Male, n (%) | | 54 (62.8%) | 33 (89.2%) | 0.002 |  | 62 (40.8%) | 57 (83.8%) | <0.001 |  | 45 (43.3%) | 51 (71.8%) | <0.001 |
| BMI, kg/m^2^ | | 25.9 ± 3.4 | 26.8 ± 2.3 | 0.19 |  | 25.3 ± 3.2 | 25.9 ± 3.0 | 0.27 |  | 24.9 ± 2.8 | 25.3 ± 3.2 | 0.37 |
| Hypertension, n (%) | | 27 (31.4%) | 20 (54.1%) | 0.01 |  | 54 (35.5%) | 34 (50.0%) | 0.02 |  | 42 (40.4%) | 37 (52.1%) | 0.26 |
| Hyperlipidemia, n (%) | | 33 (38.4%) | 20 (54.1%) | 0.17 |  | 58 (38.2%) | 34 (50.0%) | 0.15 |  | 33 (31.7%) | 30 (42.3%) | 0.10 |
| Diabetic mellitus, n (%) | | 5 (5.8%) | 9 (24.3%) | 0.01 |  | 16 (10.5%) | 15 (22.1%) | 0.03 |  | 16 (15.4%) | 12 (16.9%) | 0.97 |
| Cigarette smoking, n (%) | | 28 (32.6%) | 18 (48.6%) | 0.20 |  | 41 (27.0%) | 27 (39.7%) | 0.17 |  | 20 (19.2%) | 19 (26.8%) | 0.47 |
| Alcohol drink, n (%) | | 17 (19.8%) | 5 (13.5%) | 0.46 |  | 30 (19.7%) | 9 (13.2%) | 0.34 |  | 8 (7.7%) | 8 (11.3%) | 0.44 |
| Total cholesterol, mmol/L | | 4.70 ± 1.12 | 4.40 ± 1.60 | 0.28 |  | 4.89 ± 1.04 | 4.40 ± 1.08 | 0.004 |  | 4.8 ± 1.07 | 4.33 ± 1.04 | 0.01 |
| Triglyceride, mmol/L | | 1.53 (1.12-2.21) | 1.89 (1.36-2.70) | 0.09 |  | 1.70 (1.21-2.21) | 1.73 (1.15-3.02) | 0.42 |  | 1.25 (0.91-1.90) | 1.34 (1.04-2.05) | 0.47 |
| HDL-C, mmol/L | | 1.20 ± 0.30 | 1.07 ± 0.60 | 0.12 |  | 1.32 ± 0.52 | 1.12 ± 0.35 | 0.01 |  | 1.29 ± 0.37 | 1.22 ± 0.24 | 0.15 |
| LDL-C, mmol/L | | 2.93 ± 0.92 | 2.58 ± 1.37 | 0.13 |  | 3.00 ± 0.90 | 2.64 ± 0.92 | 0.01 |  | 2.91 ± 0.85 | 2.55 ± 0.83 | 0.01 |
| hsCRP, mg/L | | 2.10 (0.92-2.00) | 3.15 (0.84-3.99) | 0.93 |  | 1.81 (0.65-2.33) | 2.26 (0.82-2.84) | 0.48 |  | 1.97 (0.79-2.25) | 2.44 (0.82-3.00) | 0.30 |

Abbreviations: BMI, body mass index; HDL-C, high-density lipoprotein cholesterol; LDL-C, low-density lipoprotein cholesterol; hsCRP, high sensitive C-reaction protein.
