# Supplementary figures and images for: MicroRNA‐216a induces endothelial senescence and inflammation via Smad3/IκBα pathway
Source: J Cell Mol Med. 2018 Mar 7;22(5):2739–49. doi: 10.1111/jcmm.13567 (PMC5908109; doi:10.1111/jcmm.13567)

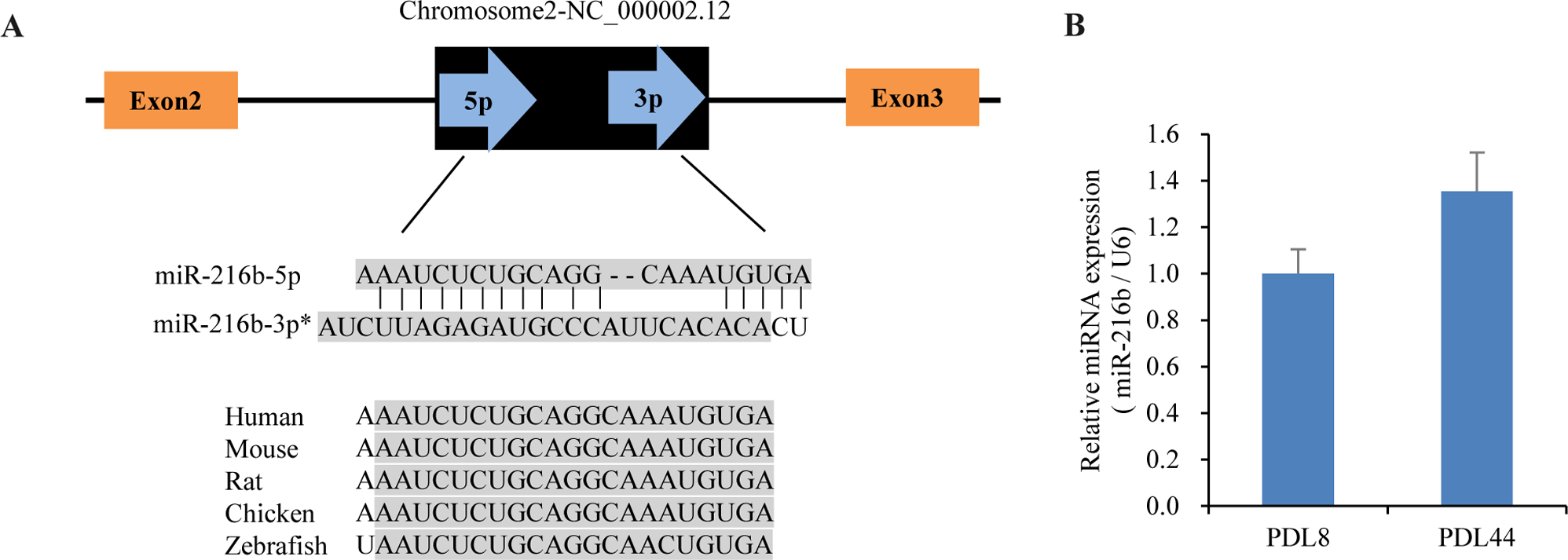

Supplement: Supplementary file 2 [file JCMM-22-2739-s002.tif]

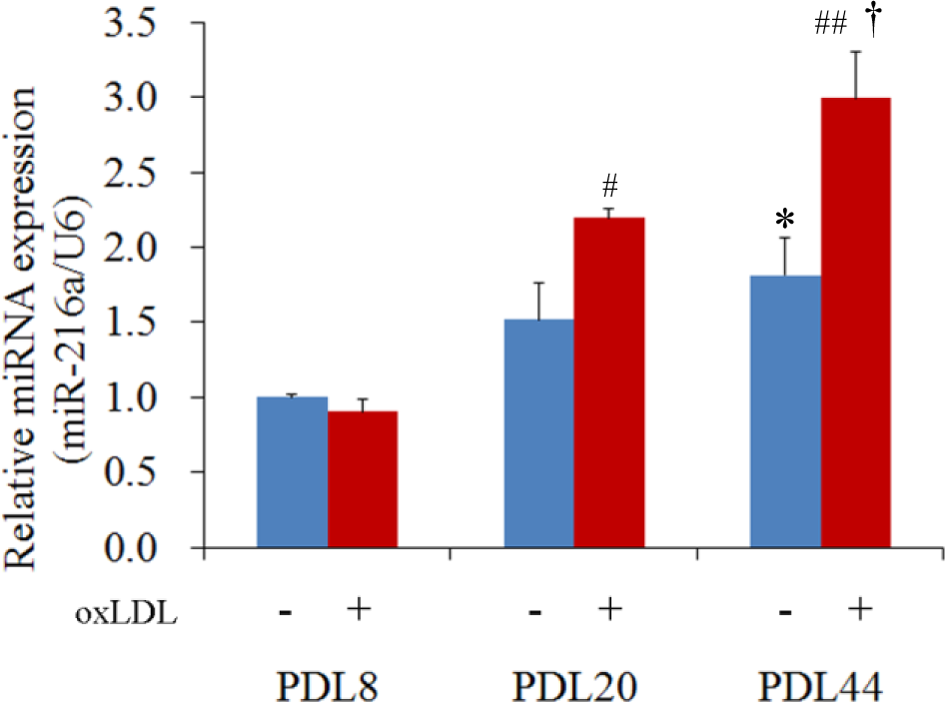

Supplement: Supplementary file 3 [file JCMM-22-2739-s003.tif]

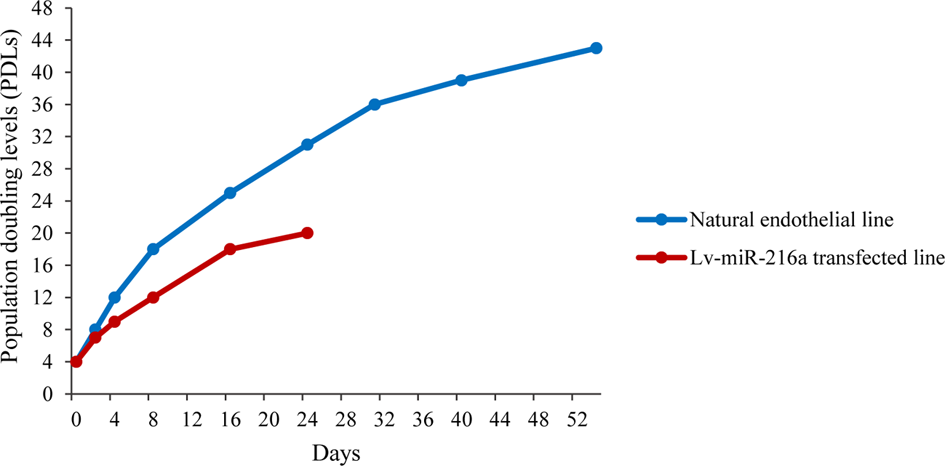

Supplement: Supplementary file 4 [file JCMM-22-2739-s004.tif]

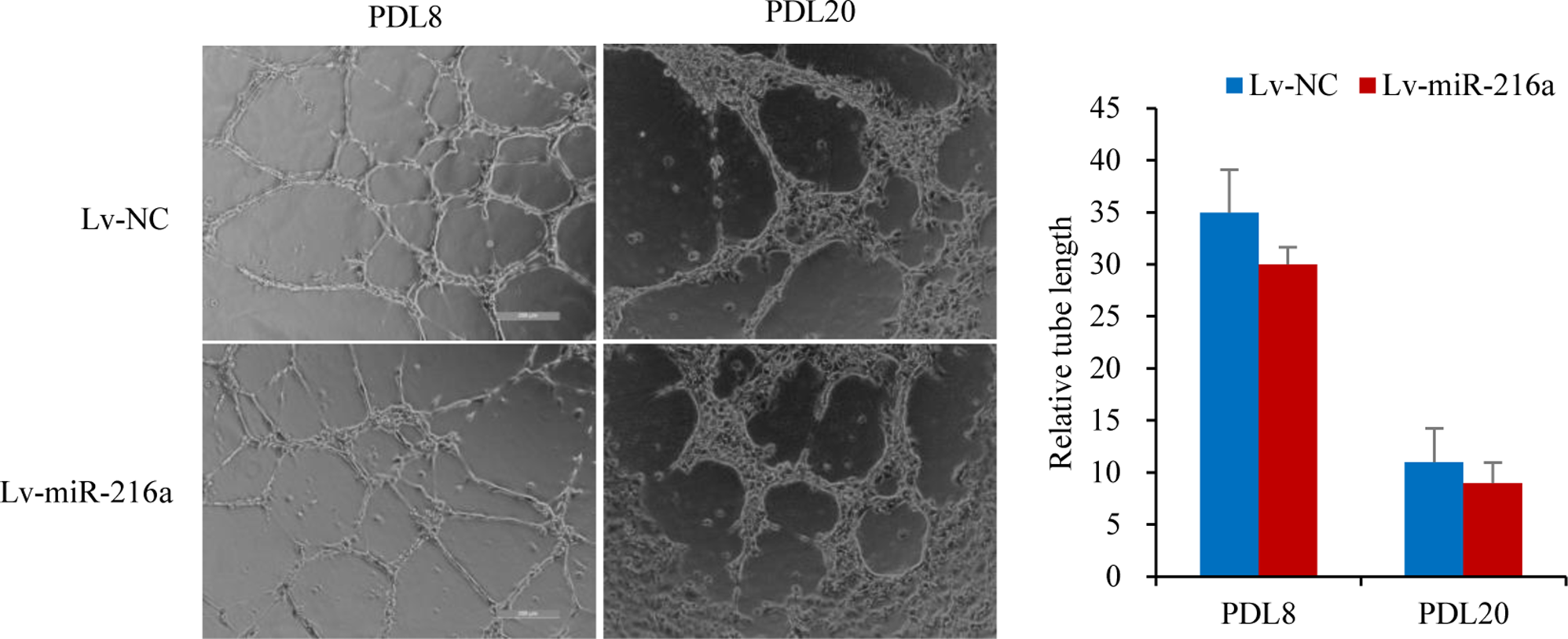

Supplement: Supplementary file 5 [file JCMM-22-2739-s005.tif]
